# Leucorrhœa, Etc.

**Published:** 1882-06

**Authors:** 


Leucorrhœa: Its Concomitant Symptoms and its Homœopathic Treatment. By A. M. Cushing, M. D. 2d edition. pp. 162. Alfred Mudge & Son. Boston. 1882.

This edition of Dr. Cushing's useful little work is in very many ways a great improvement over the former. Besides introducing new remedies, enlarging and re-writing the old, there has been added a very fair repertory, which will be especially useful. We can cordially recommend Dr. Cushing's little work to all who need assistance in prescribing for this difficult trouble.

